# Molecular docking and simulation investigation: effect of beta-sesquiphellandrene with ionic integration on SARS-CoV2 and SFTS viruses

**DOI:** 10.1186/s43141-020-00095-x

**Published:** 2020-11-27

**Authors:** Amit Joshi, G. Sunil Krishnan, Vikas Kaushik

**Affiliations:** Domain of Bioinformatics, School of Bioengineering and Biosciences, Lovely Professional University, Phagwara, Punjab India

**Keywords:** SARS-CoV2, SFTS, Beta-sesquiphellandrene, Docking, Simulation

## Abstract

**Background:**

At present, viral diseases become major concern for the world. SARS-CoV2 and SFTS viruses are deadly in nature, and there is a need for developing best treatments for them. Modern in silico approaches were found to be very handy in determining putative drug molecules. In this study, we analyze interaction of beta-sesquiphellandrene (compound belongs to ginger) with spike protein (Sp) and membrane glycoprotein polyprotein (MPp).

**Results:**

Our molecular docking and simulation study reveals the perfect binding pocket of Sp and MPp holding beta-sesquiphellandrene (bS). Binding energies for MPp-bS and Sp-bS were found to be − 9.5 kcal/mol and − 10.3 kcal/mol respectively. RMSD and RMSF values for docked complexes were found to be in selectable range, i.e., 1 to 3 Å and 1 to 8 Å respectively. Modern computational tools were used here to make this investigation fast and effective. Further, ADME analysis reveals the therapeutic validations for beta-sesquiphellandrene to act as a useful pharmacoactive compound. Beta-sesquiphellandrene provides not only inhibitory effect on spike protein of SARS-CoV2 but also similar inhibitory effects on membrane glycoprotein polyprotein complex of SFTS virus, which hampers the pathological initiation of the diseases caused by both the viruses, i.e., COVID-19 and severe fever with thrombocytopenia syndrome.

**Conclusion:**

This method of computational analysis was found to be rapid and effective, and opens new doors in the domain of in silico drug discovery. Beta-sesquiphellandrene can be used as effective medicine to control these harmful pathogens after wet lab validations.

## Background

Traditional therapy for controlling cough-cold problems with fast recovery includes mixture of ginger and jaggery. Latest studies supported such treatment strategies or home remedies for antiviral effects; it was observed that the Chinese also used same contemporary medications, like Ge Gen Tang (consist of ginger and a sweet kudzu roots) [1, 2]. Ginger holds wonderful therapeutic properties and investigated by numerous scientific researches globally, especially from India, China, and Pakistan. Ginger consists of many therapeutic chemicals, additionally been accounted in modern researches. In 1994, Dr. C V Denyer and collaborators [3] conducted 12 significant investigations on the restorative properties of ginger. A portion of these highlight its antioxidative properties, some show it to have anti-inflammatory impacts, some to its capacity to treat queasiness, a couple to its anti-emetic capacity, and there is even a paper from the West Asian area recommending that it might have a helpful impact against dementia and Alzheimer’s. Also, latest reports have explored the current proof on a few properties of ginger in wellbeing and physical movement, including its anti-malignancy properties [4]. Ginger has 6-gingerol and 6-paradol and enhances 5-fluorouracil efficiency to show anti-cancerous properties [5]. Ginger (*Zingiber officinale*) contains a very crucial anti-viral compound beta-sesquiphellandrene [6]. Prior work by Denyer and collaborators extracted compound called beta-sesquiphellandrene from ginger which holds antiviral capacity and defeats the infection caused by common cold virus [3]. Jaggery a nutritive product obtained from sugarcane holds nutritive elements sugar, calcium, iron, phosphorus, and magnesium [7].

**Fig. 1:**
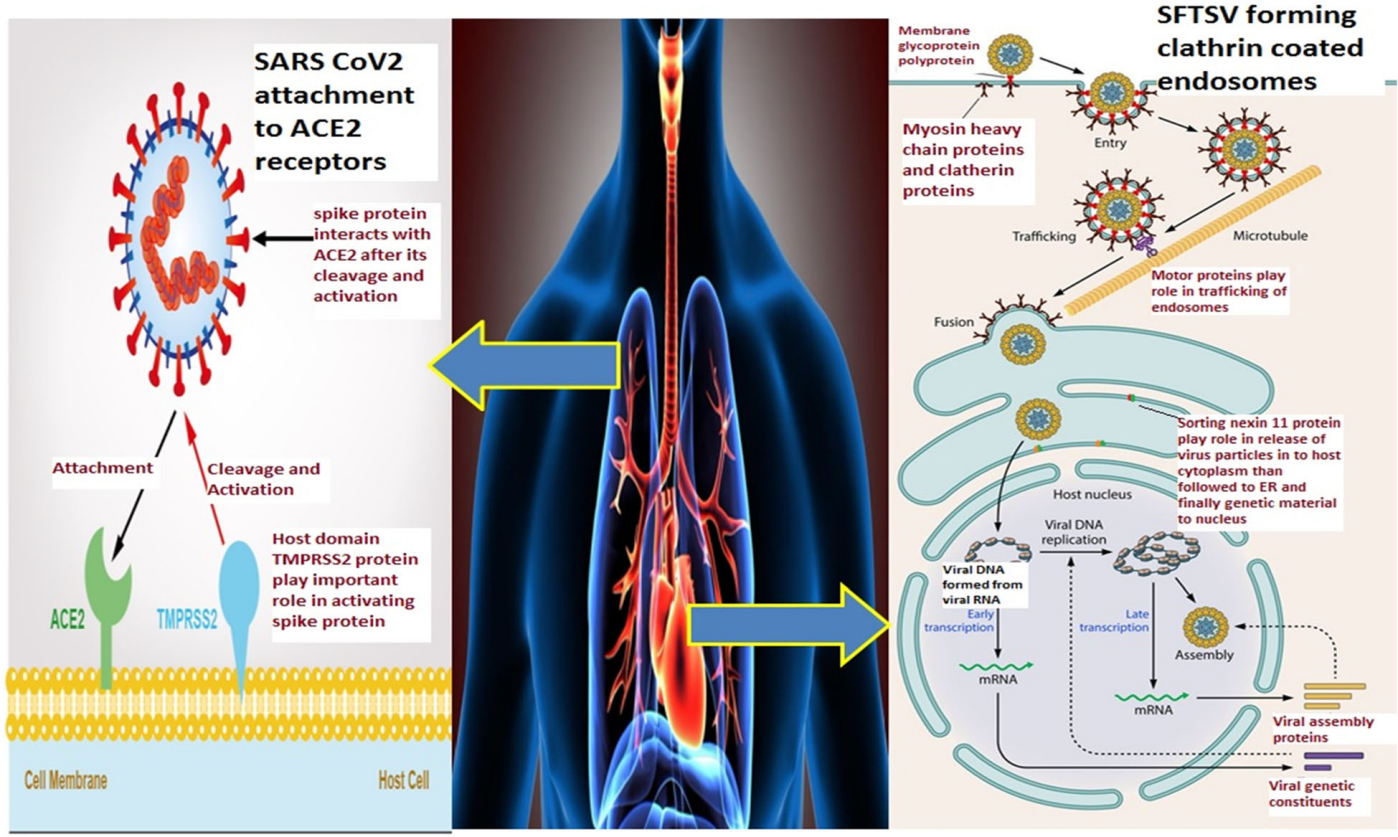
Basic pathology of viruses (host-virus interaction): SARS-CoV2 attachment with ACE2 receptors of endothelial cells and SFTSV clustered in endosomes with the help of clathrin-coated proteins

In this study, we tried to integrate ions and beta-sesquiphellandrene by in silico methods and analyze its interaction with Spike protein (Sp) [8] of SARS-CoV2 and membrane glycoprotein polyprotein (MGp) [9] of SFTS virus. Both of these proteins belong to viral domain and have a role in access to human cytological areas, particularly targeting respiratory surfaces. Spike protein plays a major role in interaction to ACE2 receptors of endothelial cells surrounding pulmonary region to bring entry of SARS-CoV2 [10], while membrane glycoprotein polyprotein plays a major role in entry to thrombocytes [11]. SFTS virus has membrane glycoprotein polyprotein complex [12], which attaches to myosin heavy chain peptides, clathrin protein, and sorting nexin-11 protein of host cellular domains. Myosin heavy chain proteins and clathrin coat proteins play a major role during internalization of SFTS viruses during endosomal formation, whereas sorting nexin-11 proteins of host side play crucial role during transfer of viral entities from endosomes to cytoplasm [13]. SFTS as well as SARS-Cov2 viruses are zoonotic in nature. SARS-CoV2 is a novel virus of family coronaviridae, which is transmitted from bats and causes COVID-19 disease. Other potential hosts that are responsible for transmitting SARS-CoV2 to humans can be categorized into wild animals and domestic animals. Wild animal’s category includes intermediate hosts like pangolins, turtles, snakes, and ferrets [14, 15]. Earlier domestic animals like poultry, dogs, and cats were thought to be underlined as intermediate hosts for SARS-CoV2 transmission, but later developments suggested that the fast antibody synthesis against SARS-CoV2 in these organisms makes them immunized from harmful consequences [14, 16]. SFTS is a novel phlebovirus of family Bunyaviridae, which is transmitted from ticks and causes severe fever with thrombocytopenia. SFTS (severe fever with thrombocytopenia syndrome) virus, thrombocytes are primary target for this virus, causes extreme deadly hemorrhagic fever [17–19]. SARS-CoV2 and COVID-19 pandemic are the most studied in recent researches and responsible for affecting the respiratory system [20]. Mode of infection for both viruses is primarily by air-droplet transmission; in Fig. 1, interaction of SARS-CoV2 and SFTS virus with human physiological systems is presented. SFTS virions target the lymphatic system, primarily spleen to target killing of monocytes and thrombocytes (platelets) [21]. SARS-CoV2 targets ACE receptors to make entry in endothelial cell [22] while SFTS virions target lymphoid tissues particularly thrombocytes [23].

Our study involves ADME analysis, molecular docking, and molecular dynamics and simulation study for investigating interaction of beta-sesquiphellandrene associated with ions to Sp of SARS-CoV2 and MGp of SFTS virus to reveal its therapeutic properties. Molecular docking studies assisted in revealing binding pockets within protein molecules where beta-sesquephellandrene can interact. To validate the interactions between ligand and protein receptors of viruses, we conducted molecular dynamic simulation studies successfully. It is very novel, fast, and effective method that can be applied for futuristic computer-based drug designing and discovery.

## Methods

### ADME analysis

ADME (adsorption, distribution, metabolism, and excretion) analysis for beta-sesquiphellandrene was conducted by using the Swiss-ADME server (http://www.swissadme.ch/) [24]. ADME analysis assisted in identifying druglikeness based on Lipinski rule of five [25]; this rule states that suitable or orally active drug must possess no more than 5 hydrogen bond donors (N–H, O–H bonds), no more than 10 hydrogen bond acceptors (all nitrogen, oxygen atoms), molecular mass less than 500 Da, an octanol-water partition coefficient, i.e., log *P* ≤ 5, and number of rotational bond should be less than 10. This rule allows best drugs to possess at least any 4 of the abovementioned characters or one can simply say that chemicals to be in selection criteria of good oral drugs are only allowed to do any single Lipinsiki violation not more than that.

### Structural retrieval

Structures for spike protein (Sp) and membrane glycoprotein polyprotein (MPp) were retrieved from the RCSB PDB server (https://www.rcsb.org/search). Drug (beta-sesquiphellandrene) structure was retrieved from the PubChem server (https://pubchem.ncbi.nlm.nih.gov/) in sdf format, and then converted to pdb format by deploying the Open Babel software [26].

### Molecular docking

Docking studies were conducted to analyze interaction between protein and drug molecules. Retrieved molecules were subjected to the PatchDock server [27] and further directed to FireDock screening. PatchDock servers are free and allow user to dock ligands with proteins (receptors), and fireDock a utility within this server assists user to screen out best possible complexes from thousands of docked complexes. It also provides ACE value (atomic contact energy) for docked complexes along with visual interpretation of interaction of ligand within binding pocket of protein. Further, the PyMOL software [28] was used to check hydrogen bonds between ligand and protein and to reveal binding pocket; this always confirms the interaction based on bond length and binding energy. Binding energies were calculated by redocking with the AutoDock Vina tool [29].

### Molecular dynamics and simulation

MD simulation was conducted by deploying GROMACS [30], a linux-based free simulation tool. MD simulation analysis of 100 ns was performed for the complex with drug and the protein molecules deploying Amber Force Field in GROMACS. The docked complexes were protonated, and counter particles (sodium ions) were added properly to make the absolute charge zero. The atoms were solvated utilizing TIP3PBOX water model with the edge of the octahedral box 10 Å away from solute particles. All through the reenactment, every complex framework is kept at the temperature of 300 K with consistent pressure. Energy minimization was accomplished for 50,000 steps.

## Results

### Beta-sesquiphellandrene: ADME analysis

**Fig. 2:**
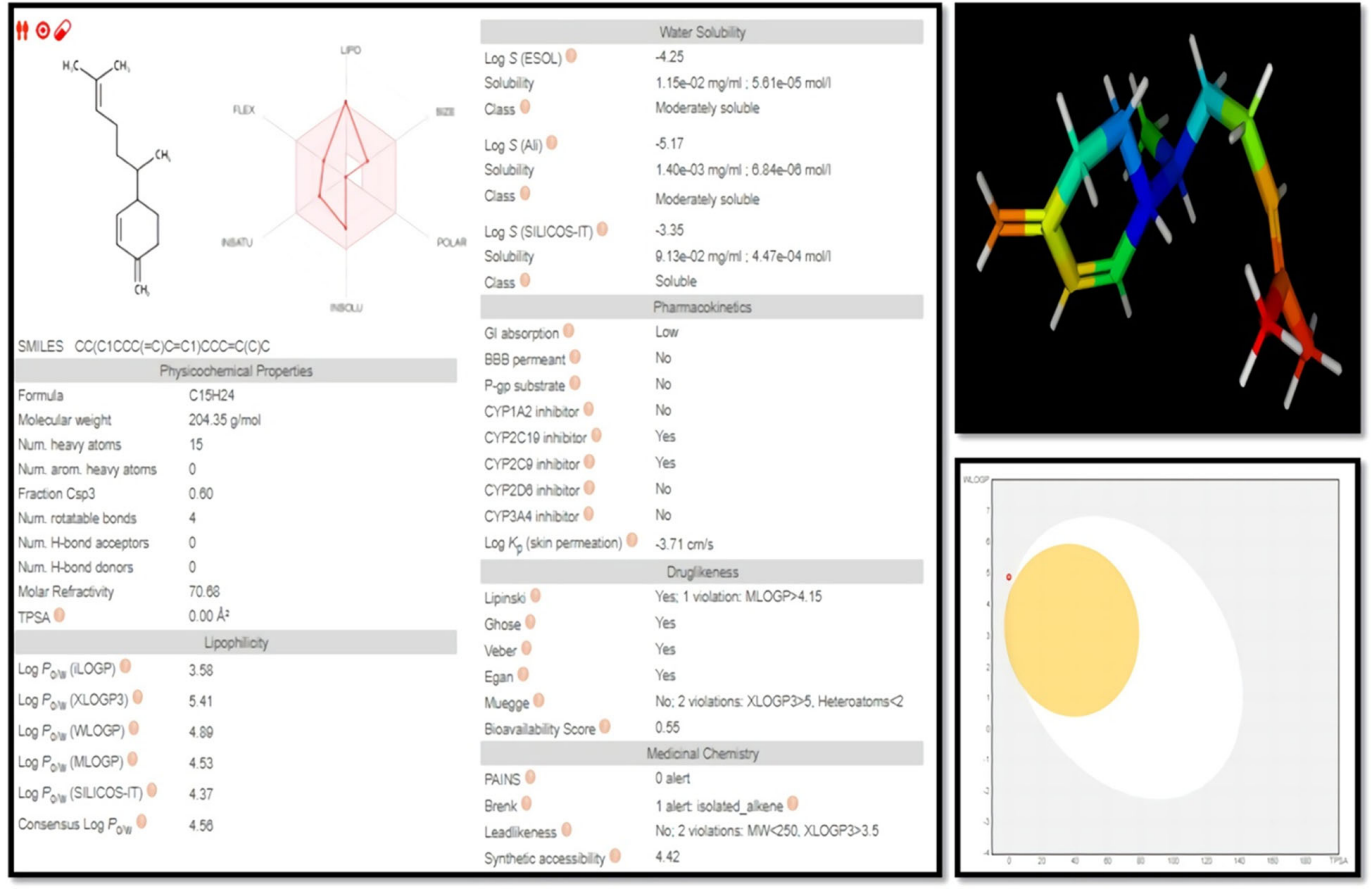
ADME analysis of beta-sesquiphellandrene along with its three dimensional structure

Beta-sesquiphellandrene ((3R)-3-[(2*S*)-6-methylhept-5-en-2-yl]-6-methylidenecyclohexene) was found to have a molecular weight of 204.35 g/mol; it does not have hydrogen bond donors and acceptors; it consists of 4 rotatable bonds, topological surface area 0Å^2^, and single Lipinski violation. Gastrointestinal absorption is low and zero blood-brain permeability. Easy to be removed during excretion as it shows interact with cytochrome p450 during xenobiotic metabolism and does not show inhibition of CYP1A2 isozyme. All characteristics reveal it to be a potent putative drug candidate. In Fig. 2, its 3D structure and all ADME properties are presented.

### Structure retrieval

**Fig. 3:**
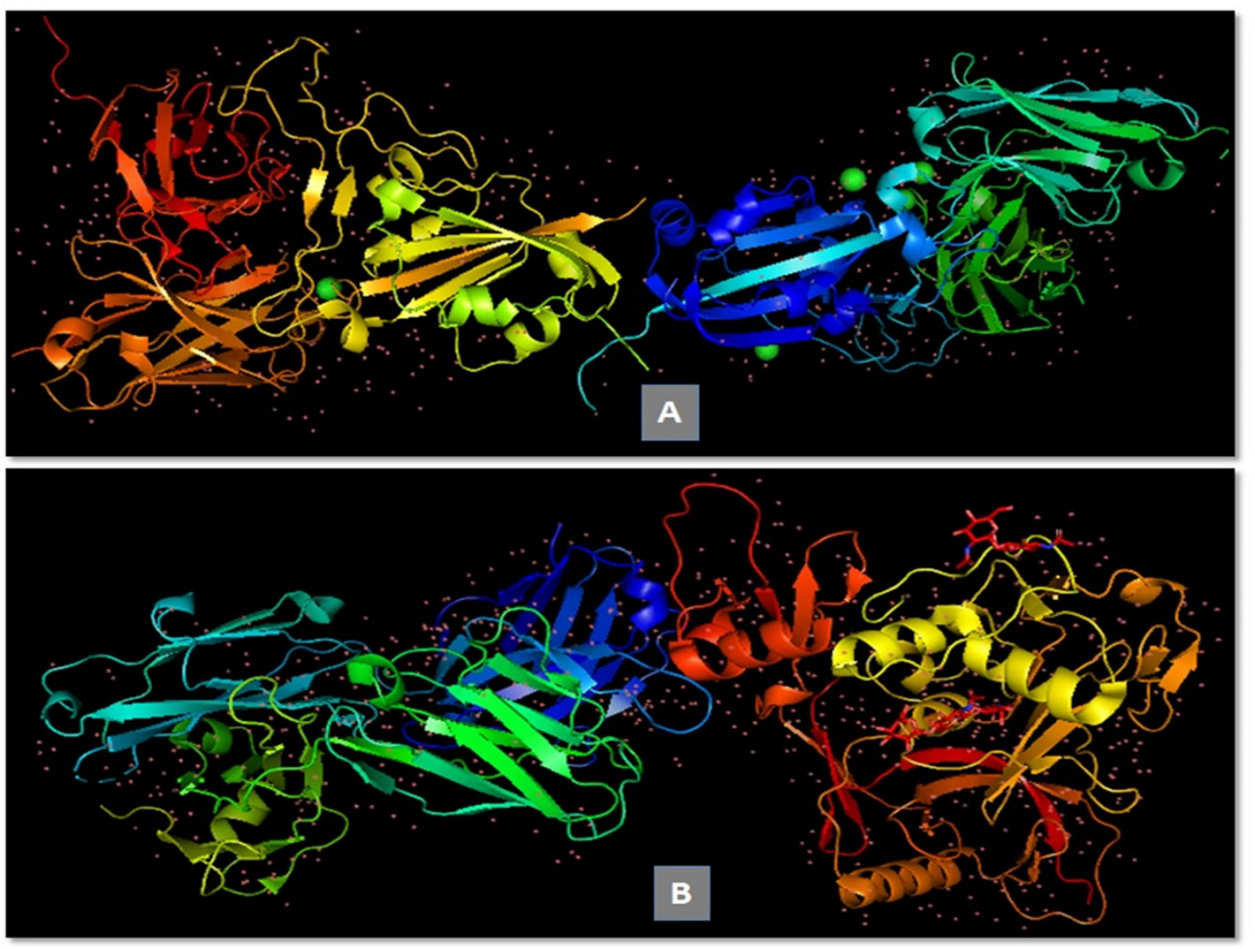
Structure of proteins. **a** Spike protein of SARS-CoV2. **b** Membrane glycoprotein polyprotein of SFTS virus

PubChem chemical ID for beta-sesquiphellandrene is 12315492, which was used to access its 3D structure in sdf format. Then, this file was converted to pdb by deploying the Open Babel tool. Structure for spike protein (Sp) and membrane glycoprotein polyprotein (MPp) was downloaded from the RCSB-PDB server. PDB ID for spike protein is 2GHW and for membrane glycoprotein polyprotein is 5Y11. Figure 3 presents the structure of both proteins.

### Molecular docking analysis

**Fig. 4:**
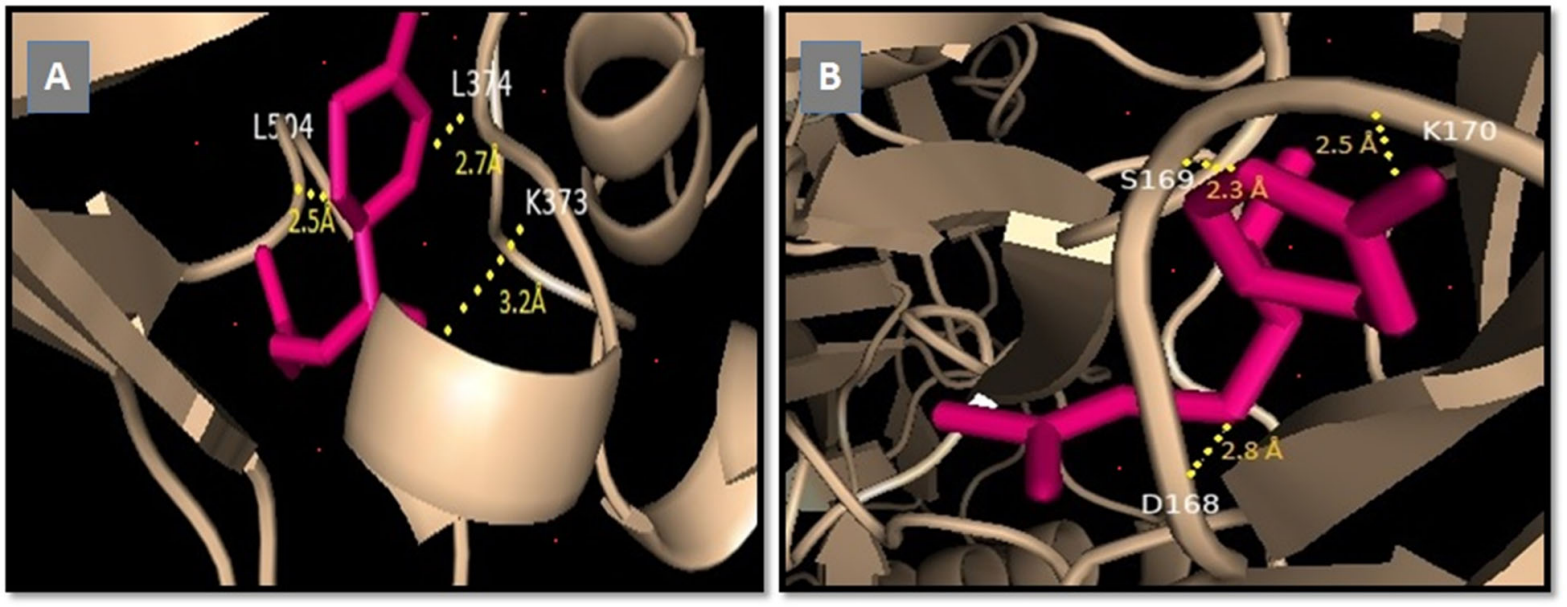
Docked models. **a** Spike protein interacting with beta-sesquiphellandrene (Sp-bS): Leu 504, Leu374, and Lys373, makes binding pocket also exhibit 2.5 Å, 2.7 Å, and 3.2 Å hydrogen bonds respectively. **b** Membrane glycoprotein polyprotein interacting with beta-sesquiphellandrene (MPp-bS): Asp168, Ser169, and Lys170, makes binding pocket also exhibit 2.8 Å, 2.3 Å, and 2.5 Å hydrogen bonds respectively. (Red dots indicate calcium, magnesium, and iron ions)

Docking studies assisted in determining ACE values for both the perfectly docked models. Membrane glycoprotein polyprotein interacting with beta-sesquiphellandrene results in complex MPp-bS, found to have ACE value − 250 and binding energy − 9.5 kcal/mol. Spike protein interacts with beta-sesquiphellandrene resulting in formation of complex Sp-bS, found to have ACE value − 265 and binding energy − 10.3 kcal/mol. Hydrogen bond interactions were also revealed during this course of investigation. In Fig. 4, both the docked models are presented, indicating hydrogen bond formation between drug molecule and protein molecule. Spike protein interacting with beta-sesquiphellandrene (Sp-bS): Leu 504, Leu374, and Lys373, makes binding pocket also exhibit 2.5 Å, 2.7 Å, and 3.2 Å hydrogen bonds respectively. Membrane glycoprotein polyprotein interacting with beta-sesquiphellandrene (MPp-bS): Asp168, Ser169, and Lys170, makes binding pocket also exhibit 2.8 Å, 2.3 Å, and 2.5 Å hydrogen bonds respectively. In Table 1, molecular docking and hydrogen bond analysis for docked models is given.

**Table 1:** Molecular docking and hydrogen bond analysis between viral proteins and beta-sesquiphellandrene molecule

Docked complex	ACE value	Binding energy (kcal/mol)	Amino acid residues of proteins/hydrogen bond length (Å)
Spike protein and beta-sesquiphellandrene (Sp-bS)	− 265	− 10.3	Leu504 (2.5 Å) Leu374 (2.7 Å) Lys343 (3.2 Å)
Membrane glycoprotein polyprotein and beta-sesquiphellandrene (MPp-bS)	− 250	− 9.5	Asp168 (2.8 Å) Ser169 (2.3 Å) Lys170 (2.5 Å)

### MD-Simulation analysis

**Fig. 5:**
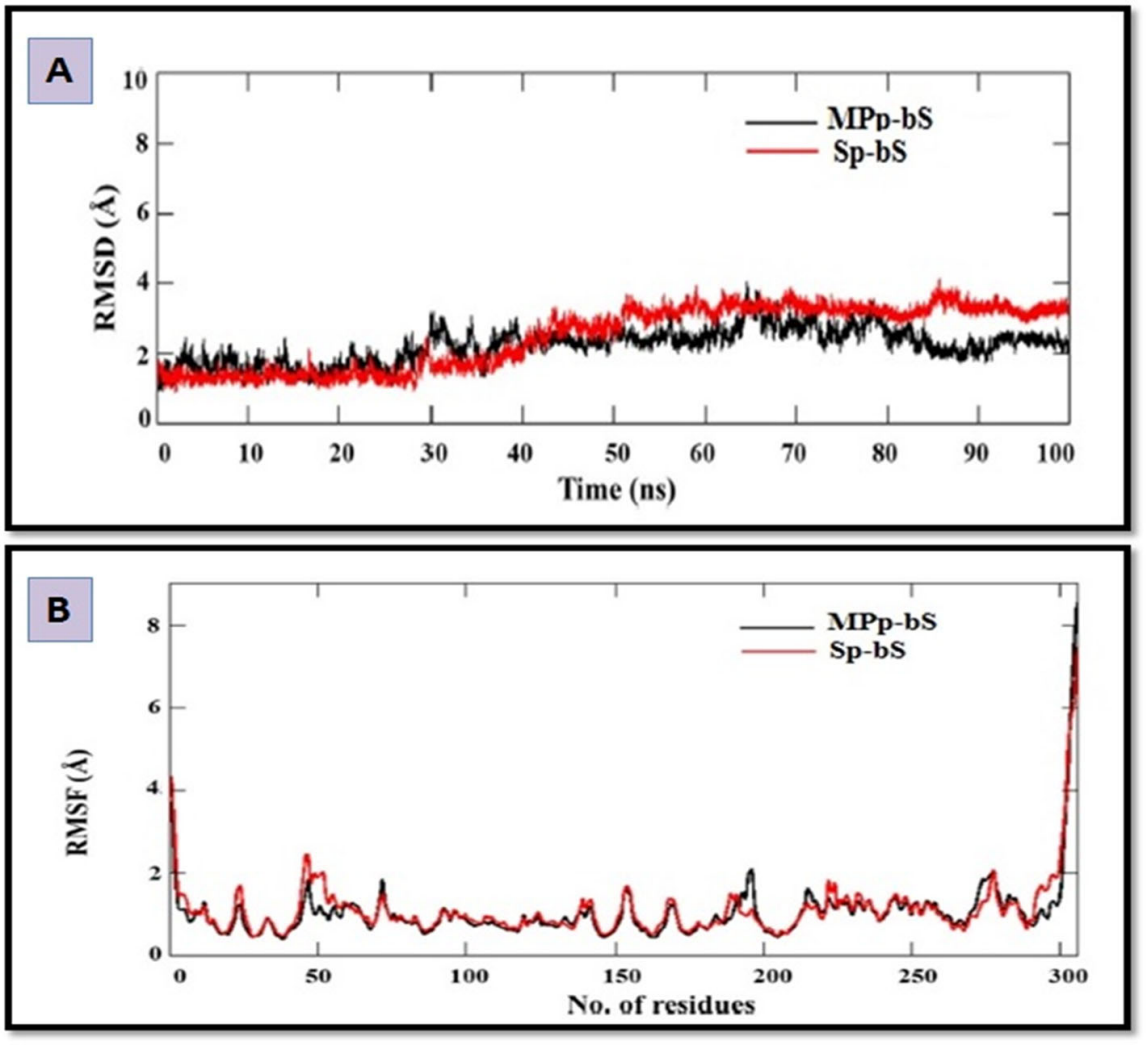
Trajectory analysis for docked complexes. **a** RMSD plot (1 to 3 Å). **b** RMSF plot (1 to 8 Å)

Molecular dynamics and simulation studies reveal stability of interactions in docked complexes. Trajectory analysis reveals root mean square deviation (RMSD) and root mean square fluctuation (RMSF) plots generated for both the docked models presented in Fig. 5. RMSD values were found to be in suitable range of 1 to 3 Å, and RMSF values were found to be in suitable range in 1 to 8 Å, for MPp-bS and Sp-bS docked complexes. An MD simulation study clearly indicates slow interaction changes in a long run of 100 ns.

MD simulation study reveals that beta-sesquephellandrene molecule can successfully interact with MPp and Sp proteins to form stable complex. Beta-sesquephellandrene is easily available as a natural food (ginger) from ancient times, and its easy availability makes it a promising source for future regimen development. This new computational approach was found to be fast and easy for drug discovery and opens new research dimensions.

## Discussion

SARS-CoV2 spike protein interacts with ACE (angiotensin-converting enzyme), to get internalized to endothelial cell. Later, SARS-CoV2 multiplies within different cells and cause not only respiratory problems but also neuropathy [31]. SFTS virus interacts with lymphoid tissue and causes immune system damages. This resulted to leukocytopenia (reduction in WBCs number) and thrombocytopenia (reduction in thrombocytes number) [32]. Beta-sesquiphellandrene was found to have perfect interaction with spike protein of SARS-CoV2 and membrane glycoprotein polyprotein of SFTS virus. SFTS virus causes asymptomatic effects like SARS-CoV2 in patients [33, 34]. SFTS virus has membrane glycoprotein polyprotein complex [12], which attaches to myosin heavy chain peptides and clathrin protein during internalization via endosomes, whereas sorting nexin-11 proteins of host side play crucial role during transfer of viral entities from endosomes to host cytoplasm [13]. Many molecular docking and molecular simulation-based studies were found to be successful in determining therapeutic peptide-based epitopes for controlling Dengue virus [35], SARS-CoV2 [10], Zika virus [36], and Whipple’s disease caused by bacterium *Tropheryma whipplei* [37]. Binding energies for MPp-bS and Sp-bS were found to be − 9.5 kcal/mol and − 10.3 kcal/mol respectively. An RMSD and RMSF value was found to be in suitable range and indicates perfectly stable interaction between ligand and proteins under consideration. Recent study on drug discovery against SARS-CoV2 for predicting drugs like remdesivir, ribavirin, sofosbuvir, galidesivir, and tenofovir were based on molecular docking and simulation-based analysis, and later, wet lab analysis makes such predictions to be fruitful in some extent [38]. Similarly, drug repurposing of lopinavir, oseltamivir, and ritonavir binding with SARS-CoV2 proteins to achieve successful treatment strategies was also based on computational analysis including docking and simulation strategies [39]. A recent study also reveals the importance of membrane glycoprotein polyprotein of SFTS virus in viral entry via membrane fusion [40]; still there is less availability of any computational-based drug discovery against this harmful virus. SARS-CoV2 started to spread from Wuhan, China, and at present caused more than 0.7 million deaths all around the globe. Ginger is a natural product and easily available to everyone for home remedy. Essential oils of medicinal importance having beta-sesquiphellandrene possess anti-viral and anti-inflammatory properties especially against SARS-CoV2 [1] and alpha-herpesvirus-1 [2]. Photochemical and antioxidants from *Zingiber officinale* are also effective against multiple ailments associated with various organ systems of the human body [41]. Murugesan et al. [42] recently revealed anti-arthritic role of *Zingiber officinale*. Molecular docking and simulation studies provide major evaluation basis for computer-based drug discovery studies [43]. Our study reveals the importance of one of the ginger compound beta-sesquiphellandrene affecting viral entry for both SARS-CoV2 and SFTS viruses which act as lifesaving in this pandemic time.

## Conclusion

Ginger, a traditional medicinal food item, contains beta-sesquiphellandrene that has potential therapeutic properties. Our study reveals that this molecule interacts and binds to spike protein of SARS-CoV2 and membrane glycoprotein polyprotein of SFTS virus to inhibit their further interaction to cells. The method of computational analysis was found to be rapid and effective and opens new doors in the domain of computational drug discovery. Beta-sesquiphellandrene can be used as effective medicine to control these harmful pathogens after wet lab validations.

## Data Availability

All data is provided in the manuscript.

## Abbreviations

SARS-CoV2: Severe acute respiratory syndrome coronavirus 2
SFTS Virus: Severe fever with thrombocytopenia virus
RMSD: Root mean square deviation
RMSF: Root mean square fluctuation
ACE value: Atomic contact energy value
ACE receptor: Angiotensin-converting enzyme
Sp: Spike protein
MPp: Membrane glycoprotein polyprotein
bS: Beta-sesquiphenalldrene
MD: Molecular dynamics
